# Causal roles of skin microbiota in skin cancers suggested by genetic study

**DOI:** 10.3389/fmicb.2024.1426807

**Published:** 2024-08-05

**Authors:** Yuhang Zhu, Wanguo Liu, Mei Wang, Xu Wang, Sibo Wang

**Affiliations:** ^1^Department of Orthopedics, China-Japan Union Hospital of Jilin University, Changchun, China; ^2^Department of Dermatology, The First Hospital of Jilin University, Changchun, China; ^3^Department of Neurology, Center for Neuroscience, The First Hospital of Jilin University, Changchun, China

**Keywords:** skin microbiota, skin cancer, Mendelian randomization, genetics, causal inference

## Abstract

**Background:**

There is evidence from observational studies that skin microbiota is linked to skin cancers. Nevertheless, the causal association between skin microbiota and skin cancers is yet to be fully clarified.

**Methods:**

A bidirectional two-sample Mendelian randomization (MR) was performed to determine the causal relationship between skin microbiota and skin cancers. A total of 294 skin microbial taxa were identified from the first genome-wide association study across three skin microenvironments of two German population cohorts. Summary data of three skin cancers (malignant melanoma, squamous cell carcinoma, and basal cell carcinoma) were obtained from the FinnGen consortium. Moreover, sensitivity analysis examined horizontal pleiotropy and heterogeneity, and microenvironment-based meta-analysis confirmed the reliability of the results.

**Results:**

We identified 65 nominal causalities and 5 strong causal associations between skin microbiota and skin cancers. Among them, the class *Bacilli* revealed a bidirectional positive relationship with malignant melanoma. The class *Betaproteobacteria* and class *Gammaproteobacteria* demonstrated a causal association with an elevated risk of malignant melanoma and basal cell carcinoma, respectively. In the reverse MR analysis, malignant melanoma was associated with a lower abundance of phylum *Bacteroidetes*. There were no indications of significant heterogeneity in instrumental variables or evidence of horizontal pleiotropy.

**Conclusion:**

Our MR analysis indicated bidirectional causal associations between skin microbiota and skin cancers, and had the potential to offer novel perspectives on the mechanistic of microbiota-facilitated carcinogenesis.

## 1 Introduction

Skin, the human body’s most extensive organ, harbors a diverse and beneficial microbial community and acts as a physical barrier to ward off pathogenic invasion (Byrd et al., 2018). The skin is mainly colonized by four bacterial phyla (*Firmicutes*, *Actinobacteria*, *Proteobacteria*, and *Bacteroidetes*), while fungi, mites, and viruses are less prevalent (Byrd et al., 2018; Chen et al., 2018). Similar to the gut microbiota, the skin microbiota is thought to participate in the development and modulation of innate and adaptive immunity and preservation of skin equilibrium (Egert et al., 2017; Harris-Tryon and Grice, 2022). In addition to facilitating immune cell maturation and differentiation, the microbiota also directly protects against pathogenic microorganisms acting as barrier and exercising ecological competition, among others (Harris-Tryon and Grice, 2022). When the barrier integrity is compromised or the commensal-pathogen equilibrium is disrupted, skin or even systemic disorders may ensue.

Skin cancers, comprising malignant melanoma (MM) and non-melanoma skin cancer (NMSC), constitute the most prevalent malignancies in Caucasians (Perez et al., 2022; Mortaja and Demehri, 2023). NMSC is predominantly composed of basal cell carcinoma (BCC) and squamous cell carcinoma (SCC), which constitute 99% of cases (Zelin et al., 2021). Several environmental factors contribute to the pathogenesis of skin cancer, including ultraviolet radiation (UV), but many others remain unknown (Lan, 2019). In light of the recent focus on microbiological components and their association with human disorder, the query arises as to how a unique microbiota may affect skin cancer susceptibility and subsequent therapeutic outcomes. As inflammation-driven carcinogenesis, persistent inflammation, and immune escape are associated with microbiological dysbiosis, it is anticipated that the microbiota is linked to the occurrence of specific malignancy (Woo et al., 2022). Similar associations have been documented, such as the involvement of *Helicobacter pylori* in gastric carcinoma and *Fusobacterium* in colorectal malignancy (Vanoli et al., 2023). Nevertheless, the connection between skin microbiota and cutaneous malignancy remains inadequately understood.

Mendelian randomization (MR) amalgamates summary information extracted from genome-wide association studies (GWAS), reduces the impact of confounding factors, and is frequently applied to elucidate potential links between exposure variables and resultant outcomes (Davey Smith and Hemani, 2014; Sekula et al., 2016). In 2022, the first GWAS exploring the genetic impact on skin microbiota among three distinct cutaneous microenvironments of German population cohorts was published (Moitinho-Silva et al., 2022). It stands in contrast to the existing comprehension of the human gut microbiota, where a diversity of related genomic loci has been detected by large GWAS (Kurilshikov et al., 2021; Rühlemann et al., 2021). The influence of gut microbiota on multiple diseases has undergone comprehensive exploration through MR analyses, but research on skin microbiota is still scarce.

In this study, we conducted a comprehensive two-sample MR analysis to evaluate the bidirectional causality between skin microbiota and three types of skin cancers (MM, SCC, and BCC) based on the FinnGen consortium (Kurki et al., 2023). This study enabled us to elucidate the function of skin microbiota in carcinogenesis, and to offer perspectives for developing novel therapeutic approaches, such as prebiotics or probiotics interventions, and microbiota transplantation.

## 2 Materials and methods

### 2.1 Data sources

Genetic variants for skin microbiota were derived from the first published genome-wide meta-analysis conducted by Moitinho-Silva et al. (2022). A sum of 1,656 skin samples was acquired from individuals within two German cohorts, KORA FF4 (*n* = 635) and PopGen (*n* = 1021). The samples were collected from three skin microenvironments, including moist skin (antecubital fossa in both cohorts), dry skin (dorsal and volar forearm in PopGen), and sebaceous skin (forehead in PopGen and retroauricular fold in KORA FF4). Microbial community patterns were obtained through sequencing the V1-V2 domains of the 16 S ribosomal RNA (rRNA) gene. Amplicon sequence variants (ASVs) in bacteria, and taxonomic groups from genus to phylum level were utilized in the GWAS. In total, 79 taxa were included in the analysis (3 phyla, 4 classes, 7 orders, 7 families, 15 genera, and 43 ASVs).

GWAS summary data for skin cancers on MM (2,993 cases, 2,87,137 controls), SCC (3,251 cases, 2,87,137 controls), and BCC (18,982 cases and 2,87,137 controls) were obtained from the FinnGen consortium, the R9 release (Kurki et al., 2023). Comprehensive information regarding the encompassed cohorts, genotypic data, endpoint specifications, and association testing can be accessed through the FinnGen webpage.

### 2.2 Instrumental variable selection

We applied the following criteria to select the instrumental variables (IVs): (1) potential IVs were identified as single nucleotide polymorphisms (SNPs) that showed an association at the locus-wide significance threshold (*P* < 1.0 × 10^–5^); (2) to ensure the independence of these variables and reduce the effect of linkage disequilibrium, a linkage disequilibrium parameter (*R*^2^) of SNP was set at 0.01, with a genetic distance of 10,000 kb; (3) SNPs with a minor allele frequency (MAF) ≤ 0.01 were removed; (4) palindromic SNPs were discarded to guarantee that the allelic effects of SNPs on the exposure matched those on the outcome during the harmonization process; and (5) IVs with an *F* statistic < 10 were excluded from the analysis to avoid the influence of weak instrument bias (Stock et al., 2002).

### 2.3 Bidirectional Mendelian randomization analysis

**FIGURE 1:**
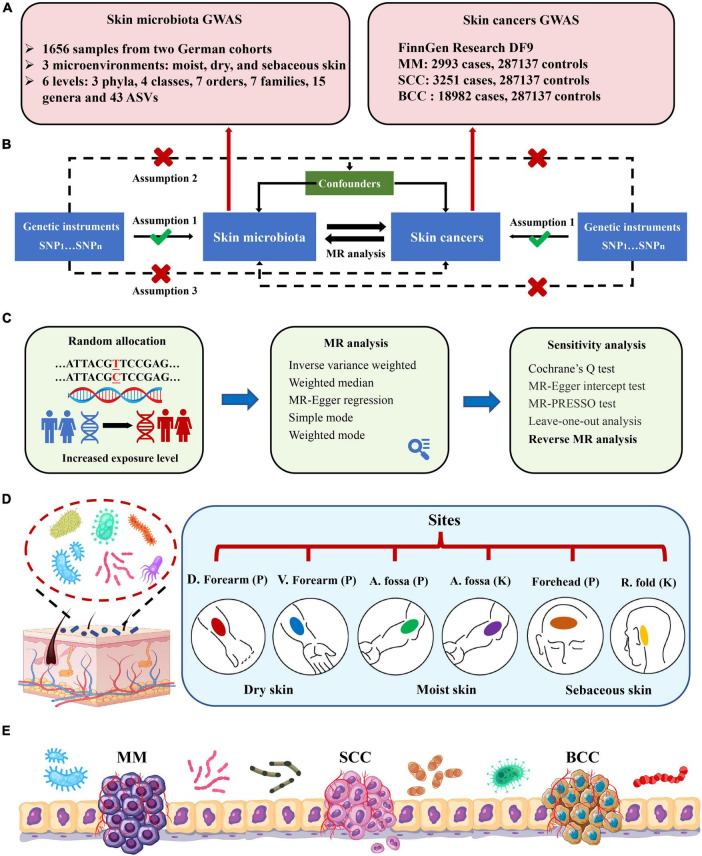
Study design and flowchart. **(A)** GWAS summary data of skin microbiota and skin cancers; **(B)** three assumptions of the current bidirectional Mendelian randomization analysis; **(C)** five MR methods and sensitivity analysis; **(D)** six skin sites from three skin microenvironments within two German cohorts. Skin sites comprise dry [dorsal (D.) forearm and volar (V.) forearm], moist [antecubital (A.) fossa], and sebaceous [forehead and retroauricular (R.) fold] microenvironments. Cohort names were shortened to PopGen (P) and KORA FF4 (K); **(E)** schema of the crosstalk between skin microbiota and skin cancers. MM, malignant melanoma; SCC, squamous cell carcinoma; BCC, basal cell carcinoma.

The MR study was structured as depicted in Figure 1. Three strict assumptions were satisfied by the genetic variations used as IVs: (1) powerful association with the exposure; (2) independence from any modifiable confounders; and (3) independence from any route linked to the outcome, other from the exposure pathway. Initially, the skin microbiota was treated as the exposure, while skin cancers were considered as the outcome. We applied five MR methods for features with multiple IVs: inverse-variance weighted (IVW) (Burgess et al., 2013), weighted median (Bowden et al., 2016), MR-Egger regression (Bowden et al., 2015), simple mode (Hemani et al., 2018a), and weighted mode (Hartwig et al., 2017). The IVW method has been shown to have more power than the others under some conditions (Hartwig et al., 2017); hence, we mainly used the IVW method for the results, and the other four methods as supplements. For a more stringent and rigorous interpretation of the causal relationship, we performed a Bonferroni correction, based on the number of bacteria within each attribute: phylum *P* = 1.67 × 10^–2^ (0.05/3), class *P* = 1.25 × 10^–2^ (0.05/4), order *P* = 7.14 × 10^–3^ (0.05/7), family *P* = 7.14 × 10^–3^ (0.05/7), genus *P* = 3.33 × 10^–3^ (0.05/15), ASV *P* = 1.16 × 10^–3^ (0.05/43). *P*-values falling within the range between 0.05 and the corrected value, were regarded as indicative of nominal significance with potential causal effect.

We used Cochran’s *Q* statistics to assess the heterogeneity of instrumental variables (Greco et al., 2015; Bowden et al., 2019). Moreover, we conducted the “leave-one-out” analysis by removing each instrumental SNP sequentially to identify potential heterogeneous SNPs. To evaluate the robustness and pleiotropy of the results, we used MR-Egger intercept tests (Bowden et al., 2015) and MR-PRESSO (Verbanck et al., 2018) to verify the existence of horizontal pleiotropy.

To investigate whether skin cancers exerted any causal influence on the identified skin microbiota, we also conducted a reverse MR analysis utilizing SNPs connected with cutaneous malignancies as IVs. The methodologies and settings employed were in line with those of the forward MR. Moreover, the Steiger directionality test was utilized to validate whether the observed causalities were biased owing to reversed causation (Hemani et al., 2017). Two-sample MR (version 0.5.6) (Hemani et al., 2018b) and MRPRESSO (version 1.0) (Verbanck et al., 2018) packages with R software (version 4.2.2) were used.

### 2.4 Microenvironment-based meta-analysis

Given the distinctiveness of skin microbiota across different microenvironments (moist, dry, and sebaceous areas), meta-analyses were carried out by combining data sets originating from the same microenvironment. The amalgamation of results occurred when the *P*-value for at least one skin site reached nominal significance (*P* < 0.05). Statistical analyses were applied using the META (version 6.5.0) (Cheung and Vijayakumar, 2016) package.

## 3 Results

### 3.1 SNP selection

In the forward MR analysis, we identified 98 SNPs associated with 15 microbial taxa for MM, 96 SNPs associated with 13 microbial taxa for SCC, and 57 SNPs associated with seven microbial taxa for BCC, following the quality control procedures. In the reverse MR analysis, we selected 72 SNPs associated with 12 microbial taxa for MM, 48 SNPs associated with 12 microbial taxa for SCC, and 290 SNPs associated with 11 microbial taxa for BCC. The *F* statistics of the IVs that showed significant associations between skin microbiota and skin cancers were all above 10, indicating that the estimates were less likely to be influenced by weak instrument bias.

In this MR analysis, the causal associations were also affected by different skin microenvironments and taxonomic levels. The majority of the causal associations occurred in the moist microenvironment (*n* = 41), trailed by dry (*n* = 19) and sebaceous (*n* = 10). A trend of increased causal correlations in more refined taxonomic levels was observed: the maximal number of causal associations was detected at the ASV level (*n* = 41), succeeded by the genus level (*n* = 10).

### 3.2 Causal associations between skin microbiota and skin cancers

#### 3.2.1 Malignant melanoma

**FIGURE 2:**
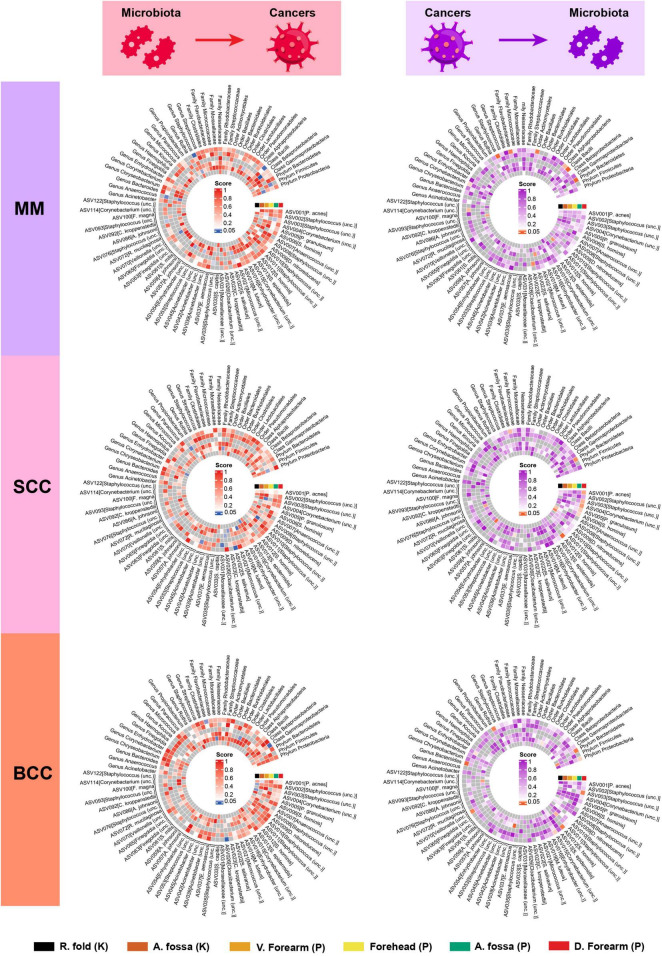
Heatmap showing bidirectional causality between skin microbiota and skin cancers. MM, malignant melanoma; SCC, squamous cell carcinoma; BCC, basal cell carcinoma.

In the forward MR analysis, we found that the phylum *Proteobacteria* at moist skin (OR = 1.07, 95% CI = 1.01–1.13, *P* = 1.60 × 10^–2^, IVW), class *Betaproteobacteria* at sebaceous skin (OR = 1.12, 95% CI = 1.05–1.19, *P* = 2.27 × 10^–4^, IVW), and class *Bacilli* at moist skin (OR = 1.06, 95% CI = 1.02–1.11, *P* = 6.99 × 10^–3^, IVW) were causally linked to MM. Furthermore, some potential causal associations were identified. The phylum *Firmicutes*, order *Clostridiales*, genus *Staphylococcus*, ASV035 [*Staphylococcus* (unc.)], ASV042 [*Acinetobacter* (unc.)], ASV054 [*Enhydrobacter* (unc.)], ASV070 [*Veillonella* (unc.)] were positively associated with MM. The genus *Anaerococcus*, ASV022 [*S salivarius*], and ASV092 [*C kroppenstedtii*] were negatively associated with MM (Figure 2 and Table 1).

**TABLE 1:** Forward MR results of the causal effects between skin microbiota and skin cancers.

Exposure	Outcome	Skin site	Microenvironment	Nsnps	OR	95% CI	*P-*value
ASV022 [*S. salivarius*]	Malignant melanoma	A. fossa (K)	Moist skin	8	0.95	0.91–1.00	3.60E−02
ASV035 [*Staphylococcus* (unc.)]	Malignant melanoma	D. Forearm (P)	Dry skin	3	1.07	1.00–1.14	4.60E−02
ASV042 [*Acinetobacter* (unc.)]	Malignant melanoma	A. fossa (K)	Moist skin	5	1.05	1.00–1.11	3.80E−02
ASV054 [*Enhydrobacter* (unc.)]	Malignant melanoma	A. fossa (K)	Moist skin	8	1.03	1.00–1.07	3.00E−02
ASV070 [*Veillonella* (unc.)]	Malignant melanoma	A. fossa (K)	Moist skin	5	1.05	1.01–1.09	1.50E−02
ASV092 [*C. kroppenstedtii*]	Malignant melanoma	D. Forearm (P)	Dry skin	7	0.96	0.93–1.00	4.00E−02
Genus *Anaerococcus*	Malignant melanoma	D. Forearm (P)	Dry skin	14	0.97	0.94–1.00	2.50E−02
Genus *Anaerococcus*	Malignant melanoma	V. Forearm (P)	Dry skin	5	0.94	0.90–0.99	1.60E−02
Genus *Staphylococcus*	Malignant melanoma	A. fossa (P)	Moist skin	6	1.08	1.02–1.15	4.70E−03
Genus *Staphylococcus*	Malignant melanoma	V. Forearm (P)	Dry skin	5	1.06	1.01–1.12	2.80E−02
Order *Clostridiales*	Malignant melanoma	A. fossa (K)	Moist skin	6	1.05	1.00–1.10	3.90E−02
Class *Bacilli*	Malignant melanoma	A. fossa (P)	Moist skin	10	1.06	1.02–1.11	2.17E−03
Class *Betaproteobacteria*	Malignant melanoma	Forehead (P)	Sebaceous skin	5	1.12	1.05–1.19	2.30E−04
Phylum *Firmicutes*	Malignant melanoma	D. Forearm (P)	Dry skin	6	1.07	1.01–1.13	1.90E−02
Phylum *Proteobacteria*	Malignant melanoma	A. fossa (K)	Moist skin	5	1.07	1.01–1.13	1.60E−02
ASV003 [*Staphylococcus* (unc.)]	Squamous cell carcinoma	A. fossa (K)	Moist skin	8	1.06	1.01–1.11	1.20E−02
ASV009 [*D. nitroreducens*]	Squamous cell carcinoma	A. fossa (K)	Moist skin	7	1.06	1.02–1.10	3.70E−03
ASV015 [*Corynebacterium* (unc.)]	Squamous cell carcinoma	R. fold (K)	Sebaceous skin	11	1.05	1.01–1.09	1.70E−02
ASV019 [*M. luteus*]	Squamous cell carcinoma	V. Forearm (P)	Dry skin	5	0.95	0.91–0.99	9.10E−03
ASV022 [*S. salivarius*]	Squamous cell carcinoma	A. fossa (P)	Moist skin	10	0.95	0.92–0.98	2.70E−03
ASV023 [*C. kroppenstedtii*]	Squamous cell carcinoma	V. Forearm (P)	Dry skin	11	1.03	1.00–1.06	2.40E−02
ASV026 [*Cloacibacterium* (unc.)]	Squamous cell carcinoma	A. fossa (K)	Moist skin	7	0.97	0.94–1.00	3.00E−02
ASV031 [*Moraxellaceae* (unc.)]	Squamous cell carcinoma	D. Forearm (P)	Dry skin	5	0.95	0.91–0.99	1.00E−02
ASV042 [*Acinetobacter* (unc.)]	Squamous cell carcinoma	D. Forearm (P)	Dry skin	7	1.04	1.01–1.08	1.80E−02
ASV070 [*Veillonella* (unc.)]	Squamous cell carcinoma	D. Forearm (P)	Dry skin	7	0.96	0.93–1.00	4.30E−02
Genus *Corynebacterium*	Squamous cell carcinoma	R. fold (K)	Sebaceous skin	2	0.88	0.79–0.98	1.60E−02
Genus *Propionibacterium*	Squamous cell carcinoma	Forehead (P)	Sebaceous skin	4	1.08	1.02–1.15	1.50E−02
Family *Rhodobacteraceae*	Squamous cell carcinoma	V. Forearm (P)	Dry skin	12	0.96	0.92–0.99	2.50E−02
ASV002 [*Staphylococcus* (unc.)]	Basal cell carcinoma	R. fold (K)	Sebaceous skin	6	1.02	1.00–1.05	2.80E−02
ASV004 [*Corynebacterium* (unc.)]	Basal cell carcinoma	R. fold (K)	Sebaceous skin	11	0.98	0.96–1.00	2.10E−02
ASV026 [*Cloacibacterium* (unc.)]	Basal cell carcinoma	A. fossa (P)	Moist skin	10	1.03	1.01–1.05	1.80E−03
Family *Flavobacteriaceae*	Basal cell carcinoma	A. fossa (P)	Moist skin	9	1.02	1.00–1.05	2.40E−02
Family *Moraxellaceae*	Basal cell carcinoma	V. Forearm (P)	Dry skin	9	1.02	1.00–1.04	1.40E−02
Order *Burkholderiales*	Basal cell carcinoma	A. fossa (K)	Moist skin	6	0.97	0.95–1.00	2.10E−02
Class Gammaproteobacteria	Basal cell carcinoma	A. fossa (P)	Moist skin	6	1.03	1.01–1.05	2.45E−03

Nsnps: the number of single nucleotide polymorphisms.

In the reverse MR analysis, we found that MM was causally linked to the phylum *Bacteroidetes* at moist skin (OR = 0.63, 95% CI = 0.46–0.86, *P* = 3.31 × 10^–3^, IVW), and class *Bacilli* at moist skin (OR = 1.62, 95% CI = 1.20–2.18, *P* = 1.39 × 10^–3^, IVW). MM was potentially positively associated with the genus *Staphylococcus*, ASV022 [*S. salivarius*], and ASV023 [*C. kroppenstedtii*], and was potentially negatively associated with the class *Alphaproteobacteria*, order *Actinomycetales*, family *Flavobacteriaceae*, genus *Corynebacterium*, ASV093 [*Staphylococcus* (unc.)], ASV114 [*Corynebacterium* (unc.)] (Figure 2 and Table 2).

**TABLE 2:** Reverse MR results of the causal effects between skin cancers and skin microbiota.

Exposure	Outcome	Skin site	Microenvironment	Nsnps	OR	95% CI	*P*-value
Malignant melanoma	ASV011 [*Staphylococcus* (unc.)]	V. Forearm (P)	Dry skin	6	0.57	0.36–0.91	2.00E−02
Malignant melanoma	ASV022 [*S. salivarius*]	A. fossa (K)	Moist skin	6	1.66	1.08–2.56	2.10E−02
Malignant melanoma	ASV023 [*C. kroppenstedtii*]	D. Forearm (P)	Dry skin	6	1.87	1.11–3.15	1.80E−02
Malignant melanoma	ASV093 [*Staphylococcus* (unc.)]	R. fold (K)	Sebaceous skin	6	0.61	0.39–0.95	2.70E−02
Malignant melanoma	ASV114 [*Corynebacterium* (unc.)]	D. Forearm (P)	Dry skin	6	0.59	0.36–0.96	3.50E−02
Malignant melanoma	Genus *Corynebacterium*	A. fossa (P)	Moist skin	6	0.74	0.55–0.99	4.60E−02
Malignant melanoma	Genus *Staphylococcus*	A. fossa (P)	Moist skin	6	1.55	1.15–2.08	3.90E−03
Malignant melanoma	Family *Flavobacteriaceae*	A. fossa (P)	Moist skin	6	0.66	0.48–0.91	1.20E−02
Malignant melanoma	Order *Actinomycetales*	A. fossa (P)	Moist skin	6	0.68	0.51–0.92	1.20E−02
Malignant melanoma	Class *Alphaproteobacteria*	A. fossa (P)	Moist skin	6	0.71	0.52–0.97	3.40E−02
Malignant melanoma	Class *Bacilli*	A. fossa (P)	Moist skin	6	1.62	1.20–2.18	1.40E−03
Malignant melanoma	Phylum *Bacteroidetes*	A. fossa (P)	Moist skin	6	0.63	0.46–0.86	3.30E−03
Squamous cell carcinoma	ASV001 [*P. acnes*]	A. fossa (P)	Moist skin	4	0.69	0.49–0.96	2.70E−02
Squamous cell carcinoma	ASV003 [*Staphylococcus* (unc.)]	A. fossa (P)	Moist skin	4	1.49	1.03–2.13	3.20E−02
Squamous cell carcinoma	ASV004 [*Corynebacterium* (unc.)]	A. fossa (P)	Moist skin	4	0.67	0.48–0.95	2.30E−02
Squamous cell carcinoma	ASV005 [*P. granulosum*]	A. fossa (P)	Moist skin	4	0.67	0.46–0.98	4.10E−02
Squamous cell carcinoma	ASV006 [*S. hominis*]	A. fossa (P)	Moist skin	4	1.52	1.01–2.29	4.50E−02
Squamous cell carcinoma	ASV007 [*Anaerococcus* (unc.)]	R. fold (K)	Sebaceous skin	4	0.61	0.39–0.97	3.60E−02
Squamous cell carcinoma	ASV008 [*Staphylococcus* (unc.)]	R. fold (K)	Sebaceous skin	4	0.62	0.42–0.90	1.30E−02
Squamous cell carcinoma	ASV012 [*S. hominis*]	A. fossa (P)	Moist skin	4	2.01	1.27–3.19	3.10E−03
Squamous cell carcinoma	ASV059 [*A. johnsonii*]	V. Forearm (P)	Dry skin	4	2.17	1.25–3.76	6.00E−03
Squamous cell carcinoma	ASV076 [*Staphylococcus* (unc.)]	V. Forearm (P)	Dry skin	4	1.79	1.07–3.01	2.70E−02
Squamous cell carcinoma	Family *Flavobacteriaceae*	A. fossa (P)	Moist skin	4	0.68	0.48–0.97	3.50E−02
Squamous cell carcinoma	Phylum *Bacteroidetes*	A. fossa (P)	Moist skin	4	0.65	0.46–0.93	1.70E−02
Basal cell carcinoma	ASV003 [*Staphylococcus* (unc.)]	A. fossa (P)	Moist skin	27	1.52	1.09–2.13	1.30E−02
Basal cell carcinoma	ASV011 [*Staphylococcus* (unc.)]	A. fossa (K)	Moist skin	27	0.67	0.47–0.97	3.30E−02
Basal cell carcinoma	ASV012 [*S. hominis*]	A. fossa (P)	Moist skin	27	1.66	1.12–2.46	1.10E−02
Basal cell carcinoma	ASV015 [*Corynebacterium* (unc.)]	A. fossa (P)	Moist skin	27	0.71	0.52–0.99	4.20E−02
Basal cell carcinoma	ASV037 [*E. aerosaccus*]	A. fossa (K)	Moist skin	27	2.12	1.31–3.44	2.40E−03
Basal cell carcinoma	ASV070 [*Veillonella* (unc.)]	A. fossa (P)	Moist skin	27	0.54	0.33–0.88	1.30E−02
Basal cell carcinoma	ASV114 [*Corynebacterium* (unc.)]	V. Forearm (P)	Dry skin	27	1.63	1.06–2.51	2.60E−02
Basal cell carcinoma	Genus *Bacteroides*	A. fossa (P)	Moist skin	20	0.5	0.31–0.81	4.50E−03
Basal cell carcinoma	Genus *Rothia*	A. fossa (P)	Moist skin	27	0.58	0.37–0.89	1.30E−02
Basal cell carcinoma	Phylum *Bacteroidetes*	A. fossa (P)	Moist skin	27	0.68	0.51–0.91	1.00E−02
Basal cell carcinoma	Phylum *Firmicutes*	R. fold (K)	Sebaceous skin	27	0.72	0.54–0.95	1.90E−02

Nsnps: the number of single nucleotide polymorphisms.

#### 3.2.2 Squamous cell carcinoma

In the forward MR analysis, the genus *Propionibacterium*, ASV003 [*Staphylococcus* (unc.)], ASV009 [*D. nitroreducens*], ASV015 [*Corynebacterium* (unc.)], ASV023 [*C. kroppenstedtii*], and ASV042 [*Acinetobacter* (unc.)] exhibited potential positive associations with SCC. Whereas, the family *Rhodobacteraceae*, genus *Corynebacterium*, ASV019 [*M. luteus*], ASV022 [*S. salivarius*], ASV026 [*Cloacibacterium* (unc.)], ASV031 [*Moraxellaceae* (unc.)], and ASV070 [*Veillonella* (unc.)] displayed potential negative associations with SCC (Figure 2 and Table 1).

In the reverse MR analysis, we found that SCC was causally linked to the phylum *Bacteroidetes* at moist skin (OR = 0.65, 95% CI = 0.46–0.93, *P* = 1.72 × 10^–2^, IVW). SCC was potentially positively associated with the ASV003 [*Staphylococcus* (unc.)], ASV006 [*S. hominis*], ASV012 [*S. hominis*], ASV059 [*A. johnsonii*], and ASV076 [*Staphylococcus* (unc.)], and was potentially negatively associated with the family *Flavobacteriaceae*, ASV001 [*P. acnes*], ASV004 [*Corynebacterium* (unc.)], ASV005 [*P. granulosum*], ASV007 [*Anaerococcus* (unc.)], ASV008 [*Staphylococcus* (unc.)] (Figure 2 and Table 2).

#### 3.2.3 Basal cell carcinoma

In the forward MR analysis, we found that the class *Gammaproteobacteria* at moist skin (OR = 1.03, 95% CI = 1.01–1.05, *P* = 5.88 × 10^–3^, IVW) was causally linked to BCC. The family *Flavobacteriaceae*, family *Moraxellaceae*, ASV026 [*Cloacibacterium* (unc.)], and ASV002 [*Staphylococcus* (unc.)] exhibited potential positive associations with BCC. Conversely, the order *Burkholderiales* and ASV004 [*Corynebacterium* (unc.)] displayed potential negative associations with BCC (Figure 2 and Table 1).

In the reverse MR analysis, we found that BCC was causally linked to the phylum *Bacteroidetes* at moist skin (OR = 0.68, 95% CI = 0.51–0.91, *P* = 1.01 × 10^–2^, IVW). BCC was potentially positively associated with the ASV003 [*Staphylococcus* (unc.)], ASV012 [*S. hominis*], ASV037 [*E. aerosaccus*], and ASV114 [*Corynebacterium* (unc.)], and was potentially negatively associated with the phylum *Firmicutes*, genus *Rothia*, genus *Bacteroides*, ASV011 [*Staphylococcus* (unc.)], ASV015 [*Corynebacterium* (unc.)], and ASV070 [*Veillonella* (unc.)] (Figure 2 and Table 2).

### 3.3 Sensitivity analysis

The causal estimates for magnitude and direction remained consistent across the weighted median, MR-Egger, weighted mode, and simple mode methods (Supplementary Tables 1, 2). No horizontal pleiotropy of the IVs was detected, as evidenced by the MR-PRESSO global test (*P* > 0.05) and MR-Egger regression (*P* > 0.05). Moreover, the Cochrane Q statistics indicated no significant heterogeneity (*P* > 0.05). The Steiger directionality test implied that the causalities identified were free of reverse causality bias (*P* < 0.05) (Supplementary Tables 3, 4).

### 3.4 Combined results from the meta-analysis

**FIGURE 3:**
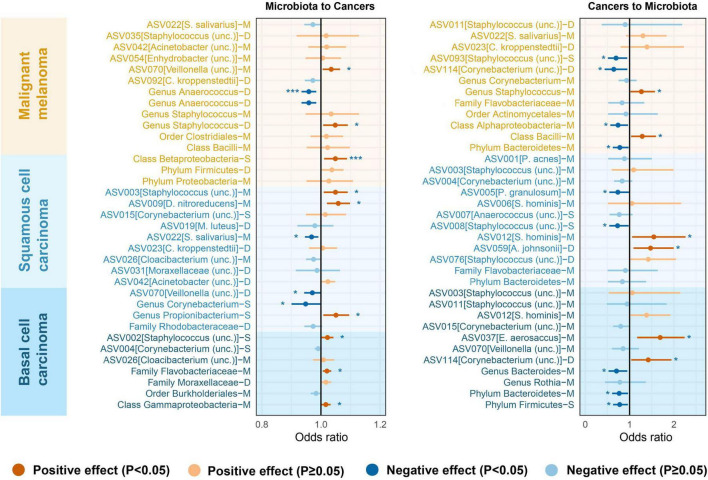
Microenvironment-based meta-analysis of bidirectional causality between skin microbiota and skin cancers. M, moist; D, dry; S, sebaceous.

To increase statistical power, meta-analyses were carried out by combining data sets originating from the same microenvironment in both cohorts. The combined results indicated that 26 bacterial taxa retained significance in the bidirectional MR analysis (*P* < 0.05). Based on the Bonferroni test, the class *Betaproteobacteria* at sebaceous skin (OR = 1.05, 95% CI = 1.01–1.09, *P* = 1.14 × 10^–2^, IVW), and genus *Anaerococcus* at dry skin (OR = 0.96, 95% CI = 0.93–0.98, *P* = 1.40 × 10^–3^, IVW) were causally associated with MM. The summary results of the meta-analysis are illustrated in Figure 3 and Supplementary Tables 5, 6.

## 4 Discussion

The benefits of the skin microbiota include establishing immune tolerance during early life, producing antimicrobial agents and immune regulatory metabolites, promoting wound healing, enhancing barrier functions, and more. Conversely, pathobionts and pathogens in the skin microbiota can lead to skin diseases (Byrd et al., 2018; Chen et al., 2018). Prior studies have indicated that microbiome dysbiosis could facilitate carcinogenesis. For instance, gut inflammation has been shown to enhance tumorigenesis by augmenting the ability of microbiota to generate genotoxins (Arthur et al., 2012). Studies have reported that some constituents of the skin microbiota may inhibit tumor expansion, and dysbiosis possibly impairs the defensive function of the microbial community (Nakatsuji et al., 2018). Moreover, it has been verified that skin microbiota potentially synthesizes cis-urocanic acid, which influences immune suppression triggered by UV exposure and impedes melanoma progression (Egert et al., 2017; Valcheva et al., 2019). In sum, several sources of evidence corroborate the notion that skin bacteria could affect tumor development.

MM is the most lethal form of cutaneous malignancy, responsible for 75% of all cutaneous malignancy-related mortalities (Woo et al., 2022). Although the contribution of the intestinal microbiota to MM has been extensively investigated lately (Spencer et al., 2021; Guardamagna et al., 2022), scarce research has been performed to elucidate the function of the skin microbiota with MM. The *Staphylococcus* encompasses various species, such as *Staphylococcus epidermidis* and *Staphylococcus aureus*, etc. Accumulating evidence discloses that *Staphylococcus* is intimately associated with the initiation and progression of multiple cancers (Wei et al., 2022). Nakatsuji et al. (2018) proposed that intravenous administration of 6-HAP originated from *S. epidermidis* can impede the expansion of melanoma cells, indicating a defensive function against MM. Conversely, Wang et al. (2018) proposed that lipoteichoic acid from *S. epidermidis* can augment the viability of melanocytes by elevating CASP5, CASP14, and TRAF1 during UV exposure. As demonstrated in our research, we discovered that the genus *Staphylococcus* was positively correlated with MM with a bidirectional causal impact. The bidirectional causal link was also observed in the class *Bacilli* to which *Staphylococcus* pertains. Nevertheless, no causal connection was detected between ASV013 [*S. epidermidis*] and MM. It is challenging to categorize all constituents of the genus *Staphylococcus* to the species rank with most amplicon sequencing methods. Therefore, the precise function of *Staphylococcus* in MM should be further clarified. Some evidence also implied that *Corynebacterium* could influence the progression of MM by an IL-17-reliant route (Ridaura et al., 2018; Mizuhashi et al., 2021). In our MR assessment, ASV092 [*C. kroppenstedtii*] exhibited a defensive impact on MM, and the genus *Corynebacterium* diminished on the skin of MM subjects. However, these associations lost significance and were inconclusively endorsed in the meta-analysis.

NMSC, the most prevalent cutaneous malignancy, is primarily constituted of SCC and BCC (Zelin et al., 2021). Literature has emphasized *Staphylococcus aureus* as a frequent risk factor for carcinogenesis (Kullander et al., 2009; Wood et al., 2018; Squarzanti et al., 2020). It has been demonstrated that a robust correlation is observed between *S. aureus* colonization and SCC. Kullander et al. (2009) detected increased colonization of *S. aureus* in biopsies and swab specimens of SCC. Wood et al. (2018) also revealed that in SCC lesional skin, *S. aureus* was the most copious bacteria in swab specimens. It was suggested that the excess of *S. aureus* in SCC could influence the hBD-2 expression, which may stimulate SCC expansion (Madhusudhan et al., 2020). In our research, ASV003 [*Staphylococcus* (unc.)] exhibited a potential causal association on promoting the progress of SCC, and SCC could lead to a higher abundance of ASV [*S. hominis*]. Besides the *Staphylococcus*, it was indicated that the *Propionibacterium* could generate coproporphyrin III, which enhanced *S. aureus* aggregation and biofilm development (Byrd et al., 2018). Furthermore, a cohort study demonstrated that the *Propionibacterium* was reduced in the skin of actinic keratosis (AK) and SCC compared to normal skin (Wood et al., 2018). It is proposed that the reduced abundance of *Propionibacterium* may be induced by the arid and flaky surface of AK, which is related to the diminished availability of sebum. The MR outcomes of our research were in agreement with the results of previous research. The genus *Propionibacterium* was regarded as a risk factor for SCC, and SCC reduced the abundance of ASV001 [*P. acnes*] and ASV005 [*P. granulosum*].

In this study, we found that *Proteobacteria* was associated with the susceptibility of skin cancers. After Bonferroni correction, phylum *Proteobacteria* and class *Betaproteobacteria* were detected to be correlated with an elevated risk of MM, and class *Gammaproteobacteria* was correlated with an elevated risk of BCC. Some bacteria belonging to *Proteobacteria* potentially increased the risk of skin cancers, including the family *Moraxellaceae*, ASV009 [*D. nitroreducens*], ASV042 [*Acinetobacter* (unc.)] and ASV054 [*Enhydrobacter* (unc.)]. The *Proteobacteria* is the largest of the bacteria and includes many opportunistic pathogens such as *Escherichia coli*, *Salmonella*, *Shigella*, *Neisseriaceae*, etc (Cosseau et al., 2016). The *Proteobacteria* could be commensal or neutral in the human body under healthy conditions, but when the cutaneous barrier is impaired, excess colonization of *Proteobacteria* is commonly observed and may induce various disorders (Skowron et al., 2021). Some researchers proposed that the *Proteobacteria* had a vital role in skin equilibrium and opportunistic infections, and elevated levels of *Proteobacteria* were observed in psoriasis subjects (Fahlén et al., 2012; Cosseau et al., 2016). In sum, *Proteobacteria* has been proposed to have a function in the progression of cutaneous malignancies, even though the particular investigation in this domain is still in its infancy. Research elucidating the function of *Proteobacteria* in skin cancers should be performed in the future.

Observational research has indicated a proclivity for a higher abundance of *Staphylococcus* and a lower presence of *Corynebacterium* in individuals with cachexia (Li et al., 2014; Herremans et al., 2019; Madhusudhan et al., 2020). In agreement with the previous studies, we also discovered that skin cancers were causally associated with cutaneous dysbiosis with more *Staphylococcus* and less *Corynebacterium* in the reverse MR assessment. It was postulated that the level of antimicrobial peptides (AMPs) in cancer cachexia subjects was higher than in normal counterparts. The *Corynebacterium* has been verified to be susceptible to the antimicrobial impact of AMPs (Percy and Gründling, 2014; Malanovic and Lohner, 2016), whereas some trials have suggested that the *Staphylococcus* has specific mechanisms that lead to resistance to AMP activity (Ryu et al., 2014; Kawada-Matsuo et al., 2021). In addition, the reverse MR outcomes implied that phylum *Bacteroidetes* remarkably diminished in subjects with skin cancers. We speculated that phylum *Bacteroidetes*, being the fewest of the four major phyla, was more vulnerable to the impacts of cutaneous dysbiosis. In brief, skin morphology is remarkably altered during the progression of malignancy and accordingly, the microbial compositions are modified. To thoroughly assess the skin microbiota, we urge further investigators to verify modifications in microbiological patterns across the spectrum of the cancer treatment process.

To the best of our knowledge, this is the first MR study to comprehensively examine the causal effect between skin microbiota and skin cancers. The bidirectional two-sample MR design that followed STROBE-MR guidelines (Skrivankova et al., 2021a,b) was performed to eliminate the disturbance of reverse causation and confounding factors. Exposure and outcome summary data were separately acquired from German and Finnish to employ nonoverlapping to evade bias. Microenvironment-based meta-analysis was carried out to increase the statistical power of the results. However, we should also take into account some limitations in our study. First, some taxa at the ASV level lacked specific species-level annotations possibly due to uncertain matches to the Ribosomal Database Project (RDP) database; furthermore, most 16S rRNA sequencing of the human microbiota has concentrated on species composition. However, recent research indicated that distinct strains of microorganisms can exert significantly different effects on the host even though they belong to the same species (Van Rossum et al., 2020). Strain-level differences have been mostly unexamined and remain a frontier for investigations of the microbiota. Second, compared to the gut microbiota, skin microbiota may be more complex due to diverse skin sites and microenvironments. Analyzing the microbiota composition across various skin sites is advantageous for elucidating the etiology of common dermatological conditions, which often exhibit a preference for particular cutaneous regions, such as psoriasis manifesting on the outer elbow and eczema occurring on the inner elbow (Kong et al., 2012; Liang et al., 2021). In this research, six skin sites were explored in the reported GWAS data, which may be insufficient to reflect the entire skin microbiota of the human body. Third, there is still just a little knowledge of the link between skin cancers and nonbacterial components of the skin microbiota, such as fungi, archaea, and viruses. Therefore, future research with more advanced sequencing technology should be performed to further explicate the effect of skin microbiota on skin cancers.

This study investigated the function of the skin microbiota in modulating skin cancers. Regarding the skin, the intestinal microbiota has been linked to several chronic inflammatory skin disorders, encompassing psoriasis, acne, atopic dermatitis, and rosacea (Woo et al., 2022). The suggested gut-skin axis (Sinha et al., 2021; Mahmud et al., 2022) may participate in the pathogenesis of cutaneous malignancy and responses to treatment. Furthermore, the onset and progression of cutaneous malignancy are affected by stimulation of skin immunity, production of microbial poisons and metabolites, barrier impairment, and UV exposure (Richardson et al., 2022; Azzimonti et al., 2023). Hence, integrating the impact of these factors will more lucidly state the function of the microbiome in cutaneous malignancy. Although the examination of prebiotics, probiotics, and microbiota transplantation still has diverse issues to address for therapeutic use, including long-term treatment safety, effectiveness, and implementation modes (Ito and Amagai, 2022, 2023), we anticipate that bacteriotherapy will grow into an attractive option for curing cutaneous diseases. The investigation of the human microbiota in cutaneous malignancy is presently ongoing. With anticipated advancements in microbiomics, we hold confidence that the intricate interactions between hosts and microbes, as well as their roles in cutaneous malignancy, will be more clearly comprehended in the future. This enhanced understanding has the potential to result in early diagnosis, preventative actions, and additional therapeutic options for skin cancers.

In summary, through bidirectional MR analysis of the causal effect between skin microbiota and skin cancers, we identified 65 nominal causalities and 5 strong causal associations. Among them, the class *Bacilli* revealed a bidirectional positive relationship with MM. Moreover, the phylum *Proteobacteria* was linked to an increased risk of skin cancers, while skin cancers were associated with a lower abundance of phylum *Bacteroidetes*. This study may offer novel perspectives on the mechanisms of skin microbiota-facilitated carcinogenesis.

## Data availability statement

The information utilized to produce the study’s findings was gathered from publicly available Genetic Consortium summary statistics. Under accession numbers GCST90133164-GCST90133313, the GWAS catalog (https://www.ebi.ac.uk/gwas) provides access to the skin microbiota summary data. Downloadable information on skin cancers in FINNGEN is available on the website (https://www.finngen.fi/en).

## Author contributions

YZ: Writing – original draft, Methodology, Investigation, Formal analysis, Data curation. WL: Writing – original draft, Methodology, Formal analysis, Data curation. MW: Writing – original draft, Visualization, Software, Resources. XW: Writing – original draft, Visualization, Validation, Software. SW: Writing – review and editing, Writing – original draft, Validation, Conceptualization.
